# The essential kinase TgGSK regulates centrosome segregation and endodyogeny in *Toxoplasma gondii*

**DOI:** 10.1128/msphere.00111-25

**Published:** 2025-03-28

**Authors:** Amanda Krueger, Sofia Horjales, Chunlin Yang, William J. Blakely, Maria E. Francia, Gustavo Arrizabalaga

**Affiliations:** 1 Department of Pharmacology and Toxicology, Indiana University School of Medicine 12250, Indianapolis, Indiana, USA; 2 Laboratory of Apicomplexan Biology, Institut Pasteur de Montevideo 123939, Montevideo, , Uruguay; 3 Department of Microbiology and Immunology, Jacobs School of Medicine and Biomedical Sciences, University at Buffalo 192705, Buffalo, New York, USA; Australian National University, Canberra, ACT, Australia

## Abstract

Intracellular replication is crucial for the success of apicomplexan parasites, including *Toxoplasma gondii*. Therefore, essential players in parasite replication represent potential targets for drug development. We have characterized TgGSK, a glycogen synthase kinase homolog that plays an important role in *Toxoplasma* endodyogeny. We have shown that TgGSK has a dynamic localization that is concurrent with the cell cycle. In non-dividing parasites, this kinase is highly concentrated in the nucleus. However, during division, TgGSK displays a cytosolic localization, with concentration foci at the centrosomes, a key organelle involved in parasite division, and the basal end. Conditional knockdown of TgGSK determined that it is essential for the completion of the lytic cycle and proper parasite division. Parasites lacking endogenous protein levels of TgGSK exhibited defects in division synchronicity and the segregation of the nucleus and apicoplast into forming daughter cells. These phenotypes are associated with defects in centrosome duplication and fission. Global phosphoproteomic analysis determined TgGSK-dependent phosphorylation of RNA-processing, basal end, and centrosome proteins. Consistent with the putative regulation of RNA-processing proteins, global transcriptomic analysis suggests that TgGSK is needed for proper splicing. Finally, we show that TgGSK interacts with GCN5b, a well-characterized acetyltransferase with roles in transcriptional control. Conversely, GCN5b chemical inhibition results in specific degradation of TgGSK. Thus, these studies reveal the involvement of TgGSK in various crucial processes, including endodyogeny and splicing, and identify acetylation as a possible mechanism by which this essential kinase is regulated.

**IMPORTANCE:**

While infection with the parasite *Toxplasma gondii* is largely asymptomatic in healthy adults, severe disease and death can result in immunocompromised individuals and in those infected congenitally. With minimal treatments for toxoplasmosis available, it is crucial to study parasite-specific processes to identify new drug targets. This study investigated the protein TgGSK, uncovering its essentiality for parasite proper division and survival. We performed an in-depth study of the functional role of this kinase. Importantly, TgGSK was shown to bear higher homology to plant proteins than its mammalian counterparts, which may allow for specific targeting of this protein.

## INTRODUCTION

*Toxoplasma gondii* is a ubiquitous parasite that can cause disease in the immunosuppressed, immunocompromised, and those infected congenitally (1–3). Many devastating consequences of an uncontrolled *Toxoplasma* infection are linked to its high replicative rate. *Toxoplasma* undergoes division through the process of endodyogeny, in which two daughter parasites gradually form within a mother cell (4, 5). This process is supported by the inner membrane complex (IMC) that, along with the plasmalemma, creates the parasite pellicle. Beneath the pellicle are 22 subpellicular microtubules that help maintain the parasite’s shape and polarity. During early endodyogeny, the centrosome duplicates, serving as an organizing center for microtubules and a docking site for dividing organelles (6). This is followed by the development of the IMCs of the daughter cells. As the IMCs of the two daughter cells grow, some organelles are made *de novo*, while the nucleus, the mitochondrion, and the plastid-like apicoplast divide between them. The mother’s IMC disappears as the two daughter parasites grow and become surrounded by the original plasmalemma. Finally, a cleavage furrow forms between the two emerging new parasites. While the steps of endodyogeny are well-characterized, the regulatory signals and proteins governing this process are not fully understood.

We recently characterized the plant-like phosphatase PPKL, which regulates daughter cell formation in *Toxoplasma* and is essential for parasite propagation. Lack of PPKL disrupts the coupling of DNA duplication and daughter cell formation and the organization of cortical microtubules without affecting centrosome splitting (7). PPKL is homologous to the *Arabidopsis* phosphatase BSU1, at the center of the plant brassinosteroid pathway (8, 9). In the absence of brassinosteroid, the phosphorylated kinase BIN2 inactivates transcription factors that regulate the expression of genes involved in processes including plant tissue growth, development, and stress responses. Brassinosteroid activates a cascade that results in the activation of BSU1, which, in turn, dephosphorylates BIN2 at a conserved tyrosine, inactivating it and leading to the transcription of brassinosteroid response genes.

*Toxoplasma* does not produce brassinosteroid, and a search for other members of this well-characterized plant signaling pathway did not reveal clear homologs except for TGGT1_265330, a serine/threonine kinase member of the glycogen synthase kinase 3 beta subfamily (GSK3). Herein, we refer to it as TgGSK. GSKs are multifunctional kinases that influence a broad range of cellular functions. In *Arabidopsis*, an analysis of BIN2 interactors found over 400 proximal proteins in over 15 different clusters with different biological functions, with 267 of these being BIN2 interactors or their substrates (10). Similarly, other members of the GSK family are known for having a wide range of functions, and accordingly, their disruption leads to pleiotropic effects.

A previous study, which referred to TgGSK as TPK3, indicated that it bears 54% homology to GSKs over the catalytic domain and showed that the recombinant protein has kinase activity and can autophosphorylate (11). Nonetheless, the localization or function of this *Toxoplasma* kinase was not investigated. Here, we report that TgGSK is an essential kinase in *Toxoplasma* that displays a varying localization dependent on parasite division. Concordantly with its expected pleiotropy, it influences a broad range of critical functions. Knockdown of TgGSK causes abnormal division phenotypes, including defects in the centrosome, apicoplast, and nuclear segregation. Furthermore, phosphoproteome and transcriptome analyses suggest a role in splicing for TgGSK. Finally, we show that TgGSK forms a complex with the acetyltransferase GCN5b and that the inhibition of GCN5b acetyltransferase activity leads to TgGSK degradation. In sum, our work characterizes an essential kinase that regulates a broad range of critical functions in the human pathogen *Toxoplasma gondii*, uncovering a potential target for therapeutic intervention.

## RESULTS

### *Toxoplasma* GSK is related to plant kinases

**Fig 1:**
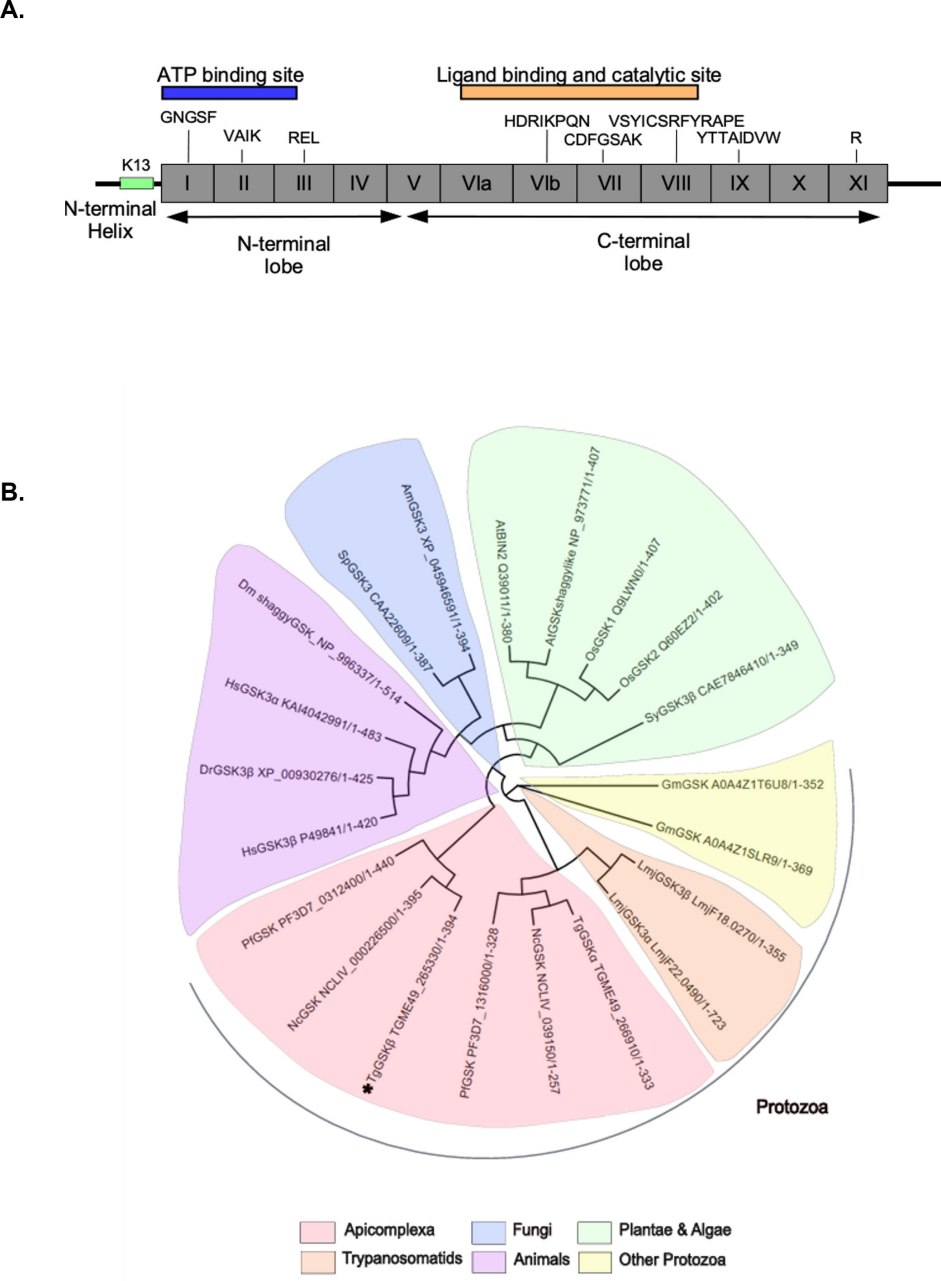
TgGSK is a GSKβ closely related to other apicomplexan and protozoan GSKs. (A) Graphical representation of the TgGSK protein, showing the relative positions of the 12 conserved ePK motifs (indicated in roman letters). A selection of highly conserved residues is labeled. The ATP-binding domain is represented in blue, and the ligand binding site and catalytic site in orange. The conserved acetylation site (K13) is located in the extra N-terminal α-helix shown in green. (C) Phylogenetic tree including 21 GSKs from different organism representative of all eukarya kingdoms; At, *Arabidopsis thaliana*; Oz, *Oryza sativa Japonica*; Sy, *Symbiodinium* sp.; Sp, *Schizosaccharomyces pombe*; Am, *Aspergillus melleus*; Dm, *Drosophila melanogaster*; HS, *Homo sapiens*; Dr, *Danio rerio*; Lm, *Leishmania major*; Gm, *Giardia muris*; Nc, *Neospora caninum*; Pf, *Plasmodium falciparum*; Tg, *Toxoplasma gondii*. TgGSK marked by an asterisk.

TGGT1_265300 codes for a 394 amino acid serine/threonine eukaryotic protein kinase (ePK) composed of 12 highly conserved motifs (12). It is a member of the subfamily GSK, defined by the presence of conserved CDFGSAK and SYICSR motifs (highlighted in red, Fig. 1A) (13). The signature sequence motifs associated with the ATP-binding site, the ligand-binding site, and the catalytic domain are conserved across all GSKs (Fig. 1A). Existing phosphoproteomic data (14) show that TgGSK is phosphorylated at tyrosine 211 (Fig. 1A), which is conserved throughout the GSK family and equivalent to the tyrosine regulated by BSU1 in the plant GSK BIN2 (9). A Clustal Omega alignment with 21 sequences of different reference organisms shows that TgGSK and *Plasmodium* GSK cluster together with other protozoan GSKs. TgGSK appears more closely related to a cluster of plant kinases, including *Arabidopsis* BIN2, than to their animal and fungi counterparts (Fig. 1B).

### TgGSK has a dynamic localization that is cell cycle dependent

**Fig 2:**
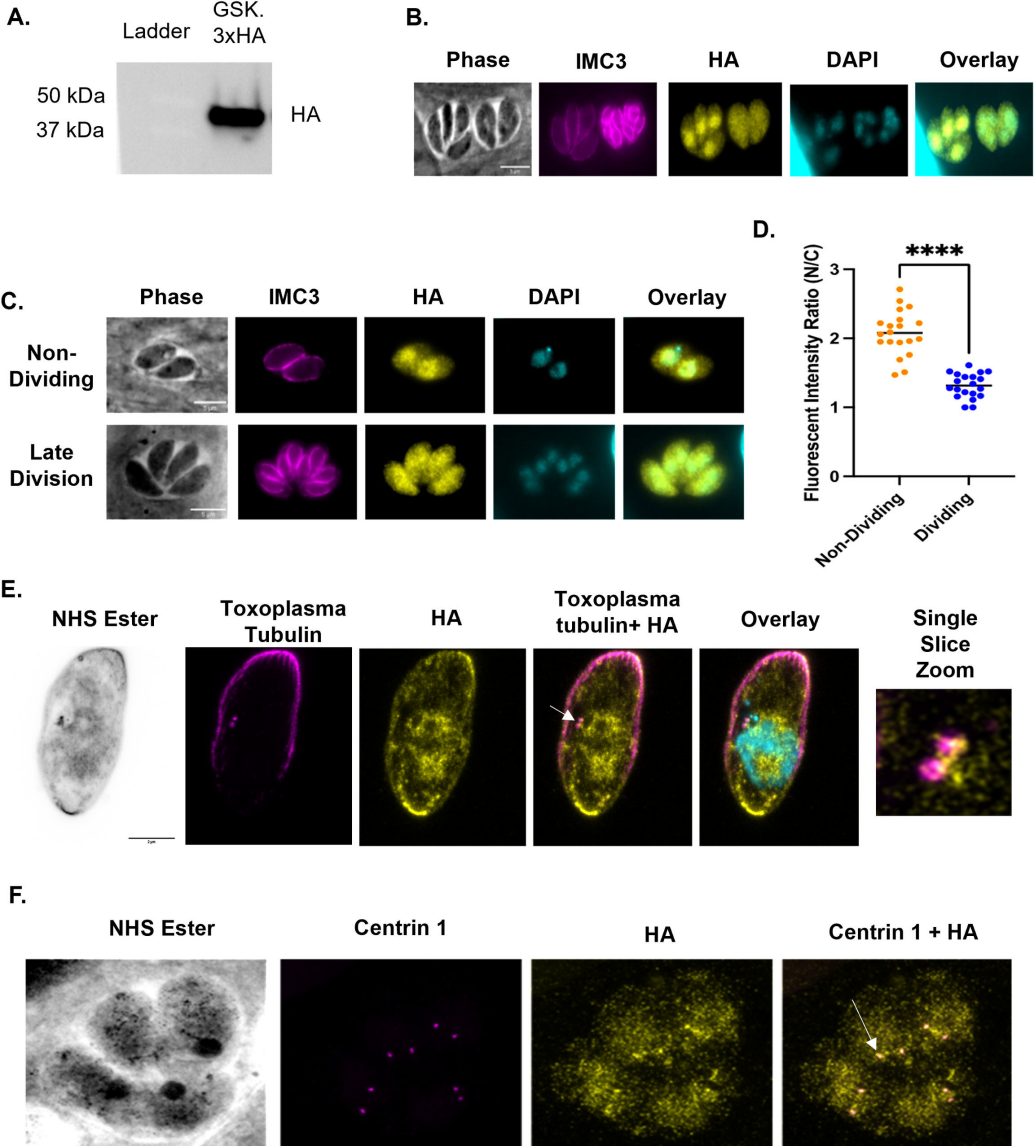
TgGSK has a dynamic, cell cycle-dependent localization. (A) Western blot of protein extract from parasites in which the endogenous TgGSK includes a 3×HA epitope tag. Blots were stained for HA (top) and Sag 1 (bottom). (B, C). Parasites of the TgGSK.3xHA strain were grown intracellularly for 24 hours before performing IFA using antibodies against HA and IMC3, and the DNA stain DAPI. In panel C, parasites in the top panels are not dividing, while those in the bottom panel are undergoing division. (D) Quantification of the fluorescent intensity ratio of nucleus to cytosol in non-dividing and dividing parasites; *n* = 20 per condition, *P* < 0.0005. (E) Expansion microscopy of a non-dividing parasite staining for *Toxoplasma* tubulin (magenta), HA (yellow), and DRAQ5 (cyan, color in overlay) to visualize parasite structure and centrosomes, TgGSK, and nuclear material, respectively. A single-slice zoomed image of the centrosomes is shown to closer visualize TgGSK localization to this organelle. (F) Expansion microscopy of four dividing parasites in a vacuole staining for centrin 1 and HA to visualize TgGSK’s colocalization with the centrosomes. Arrowhead shows an example of TgGSK colocalized with a centrosome.

To determine TgGSK’s localization, we used CRISPR/Cas9 to create a strain in which the endogenous protein includes a triple hemagglutinin tag (GSK.3xHA). Western blot analysis shows a protein of approximately 44 kDa, which matches the expected molecular weight (Fig. 2A). Immunofluorescence assays (IFA) probing for HA and for IMC3, which allows us to distinguish between dividing and non-dividing parasites, shows that in non-dividing parasites, TgGSK is primarily in the nucleus (Fig. 2B and C). In contrast, in dividing parasites, it was seemingly throughout the cell (Fig. 2B and C). To quantify this, we analyzed the HA signal intensity in 20 non-dividing and 20 dividing parasites, using ImageJ. As expected, non-dividing parasites showed a higher nuclear-to-cytosolic signal ratio (Fig. 2D).

Next, we performed ultrastructure expansion microscopy (UExM) for higher resolution analysis. We were able to resolve TgGSK’s cytosolic localization to areas of tubulin concentration, which are reminiscent of centrosomes. In addition, TgGSK localized to the basal and apical ends of the parasites, with a higher concentration in the basal end (Fig. 2E). Co-staining with centrin 1 confirmed that TgGSK concentrates around the centrosomes in both non-dividing and dividing parasites (Fig. 2F). In sum, IFA and UExM analyses show that TgGSK has a dynamic localization dependent on the division stage. Since it is known that centrosomes play a key role in parasite division, we decided to focus on this localization moving forward.

### TgGSK is essential for parasite survival

**Fig 3:**
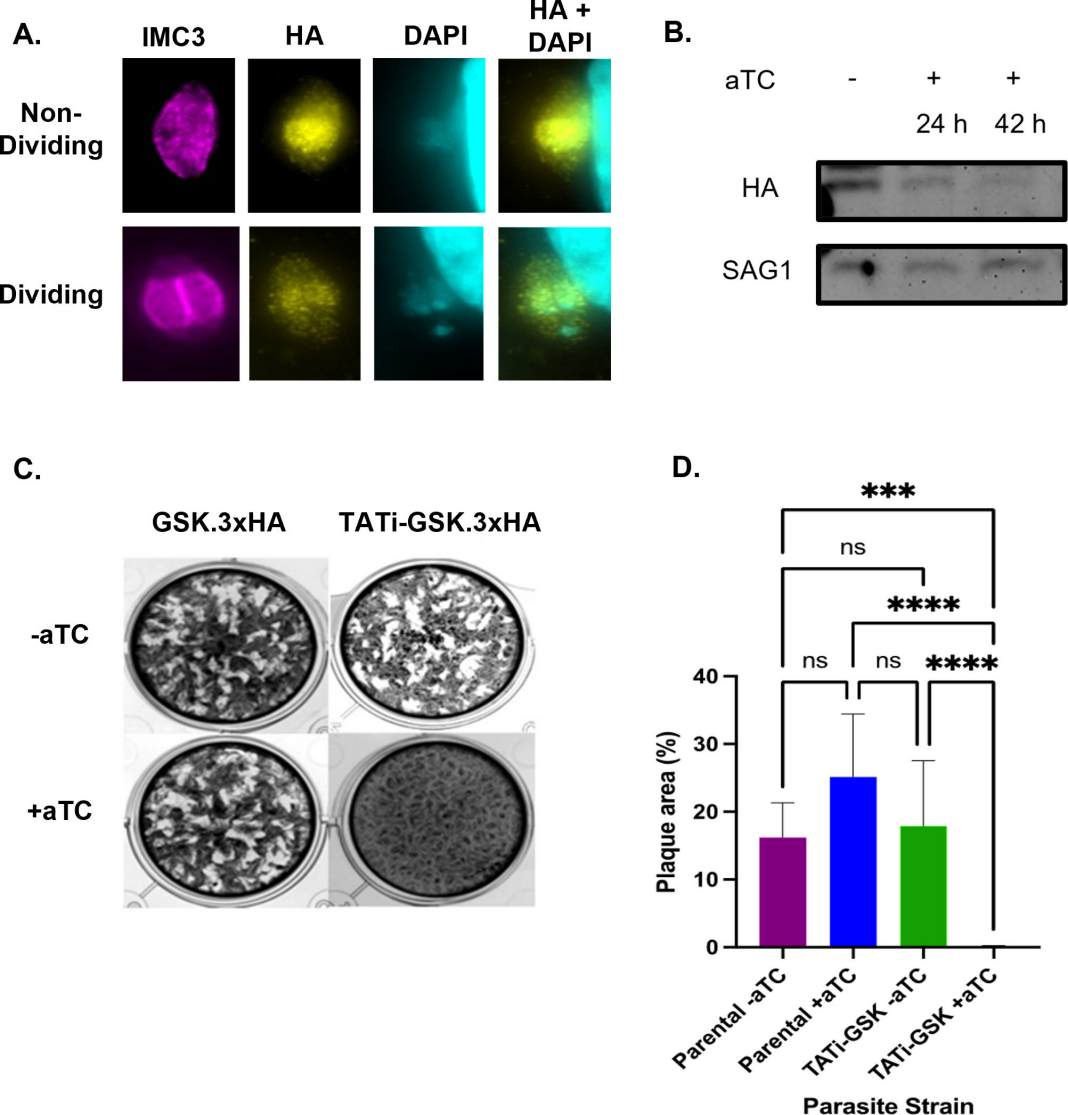
TgGSK is essential for propagation in tissue culture. (A) Parasites of the TATi-GSK.3xHA strain were grown in culture for 24 hours to perform IFA with antibodies against IMC3 and HA and the DNA stain DAPI without the addition of aTC. (B) Western blot of protein extract from TATi-GSK.3xHA parasites grown in no aTC (−) or in aTC for 24, 42, or 72 hours. Blots were probed for HA to monitor TgGSK or Sag1 as loading control. (C) Plaque assay of parental (GSK.3xHA) and TATi-GSK.3xHA knockdown parasites incubated for 6 days with or without aTC. (D) Quantification of plaque assay. ****P < 0.0005, ***P < 0.005, ns: no significance, *n* = 9 wells per condition (three biological replicates with three experimental replicates each).

In a *Toxoplasma* genome-wide CRISPR screen, TgGSK was assigned a fitness value of −4.12 (15), suggesting that this protein is essential. Accordingly, we generated a conditional mutant strain by replacing the TgGSK promoter with a tetracycline-regulatable promoter (TATi) (16) . IFA showed that TgGSK’s localization in the resulting strain (TATi-GSK.3xHA) is as expected (Fig. 3A). Western blot shows that TgGSK expression is significantly reduced after 42 hours of treatment with the tetracycline analog aTC, with near complete lack of protein at 72 hours (Fig. 3B). Importantly, TATi-GSK.3xHA parasites failed to propagate and form plaques in the presence of aTC, confirming that TgGSK is essential (Fig. 3C and D).

### Knockdown of TgGSK causes abnormal division phenotypes

**Fig 4:**
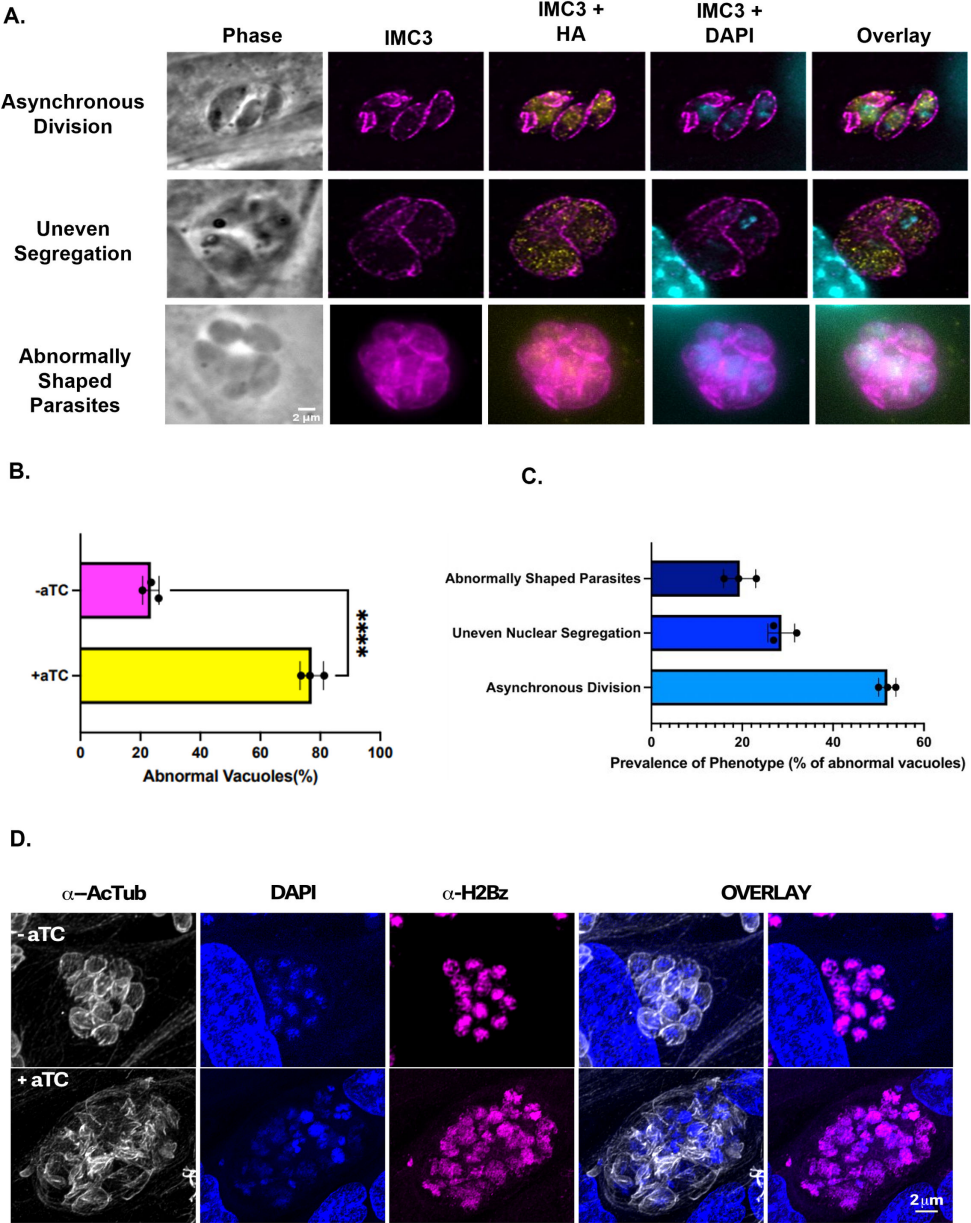
Knockdown of TgGSK causes abnormal division phenotypes. (A) IFA of TgGSK knockdown parasites after 42 hours of incubation with aTC. IMC3, HA, and DAPI staining visualize mother and daughter cell IMC, TgGSK, and nuclear material, respectively. Representatives of the main phenotypes observed are shown in panel B. Quantification of the percentage of normal vacuoles in TATi-GSK parasites with and without 42 hour incubation with aTC. *N* = 3, 100 total parasites over the replicates. The error percentage shown is the standard deviation. (C) Quantification of the different division phenotypes seen after 42 hours of TgGSK knockdown. (D) IFA showing division phenotypes of TgGSK knockdown parasites after 96 hours of incubation with aTC. Acetylated tubulin, DAPI, and H2Bz staining visualize parasite structure, nuclear material, and histones, respectively. Arrowheads point to diffuse nuclear material and H2 histone.

To explore the impact of TgGSK’s loss on cell division, we performed IFAs on the TATi-GSK.3xHA strain grown with and without aTC. After 42 hours of aTC exposure, staining for IMC3 revealed significant division defects despite some residual TgGSK (Fig. 4A). The most common phenotypes observed include asynchronous division, incomplete nuclear segregation, and vacuoles with parasites of abnormal shape (Fig. 4A). Quantification of 100 vacuoles over three experimental replicates showed that, while 76.5% ± 2.2% of parasites appear to divide normally in the absence of aTC, only 22.9% ± 3.18% of parasites exhibit normal division in its presence (Fig. 4B). Among the vacuoles grown with aTC that show aberrant division, 51.9% ± 1.5% exhibit asynchronous division, 28.6% ± 2.4% uneven segregation, and 19.4% ± 2.9% abnormally shaped parasites (Fig. 4C). After 96 hours in aTC, all phenotypes became more pronounced, with parasites continuing to divide unsuccessfully (Fig. 4D). Significant structural defects included poorly organized tubulin and impaired nuclear segregation, as well as diffuse staining for the histone marker H2, indicating effects on chromatin condensation (Fig. 4D). Overall, analysis of the TgGSK knockdown strain reveals a role for the kinase in parasite division.

### TgGSK knockdown causes centrosome abnormalities

**Fig 5:**
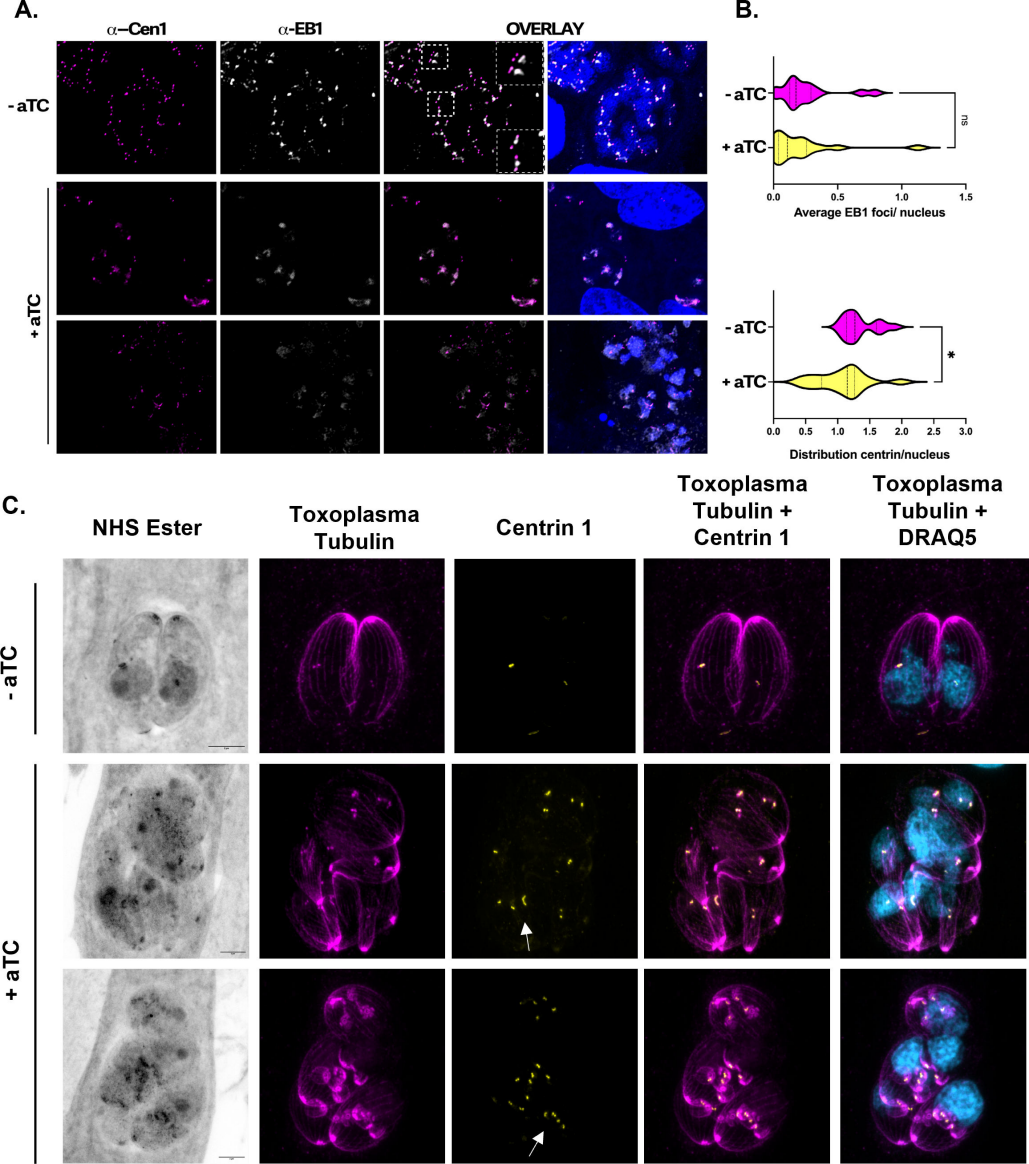
TgGSK knockdown parasites have a centrosome duplication defect. (A) IFA TATi-GSK parasites treated with TC for 96 hours stained for centrin 1 (centrosomes) and EB1 (mitotic spindles). Outlined boxes show parasites with abnormally dividing centrosomes. (B) Quantification of centrin and EB1 signal in panel A measuring distribution of signal per parasite nucleus. *N* = 3 replicates with 100 vacuoles quantified. Statistical analysis: two-tailed *t*-test with Welsh’s correction. *P < 0.05, ns: no significance. (C) Expansion microscopy of TATi-GSK parasites treated with TC for 42 hours. Arrows indicate elongated centrosomes that seem unable to undergo fission.

Since we detected TgGSK at the centrosomes and observed nuclear segregation defects in TgGSK knockdown parasites, we monitored the centrosome in the mutant parasites. Indeed, IFA using centrin 1 and the mitotic spindle marker EB1 revealed abnormalities in centrosome morphology, including elongated centrosomes and incomplete fission (Fig. 5A). We quantified the distribution of EB1 and centrin per parasite nucleus using ImageJ. In normal cultures, the average amount of centrosomes per nucleus should be around 1.25, with non-dividing parasites having one centrosome and dividing parasites having two (17). While the number of nuclei displaying EB1 foci was similar between control and TgGSK knockdown parasites, the average number of centrosomes per nucleus was significantly lower in aTC-treated parasites. On average, untreated parasites displayed 1.33 centrosomes/nucleus, while aTC-treated parasites displayed an average of 1.07. Strikingly, a significant proportion of vacuoles displayed a single or no centrosome and multiple nuclei, with some vacuoles showing only one centrosome for four nuclei (Fig. 5B). Importantly, UExM confirmed the various phenotypes observed by IFA (e.g., abnormal parasite structure and nuclear segregation defects) and highlighted the abnormally shaped centrosomes at a higher resolution (Fig. 5C). We also observe an excess in daughter parasite buds (Fig. 5C). Overall, these results suggest that TgGSK knockdown causes centrosome overduplication in some cells, leading to an excess of daughter cell budding.

### Knockdown of TGGSK affects apicoplast division

**Fig 6:**
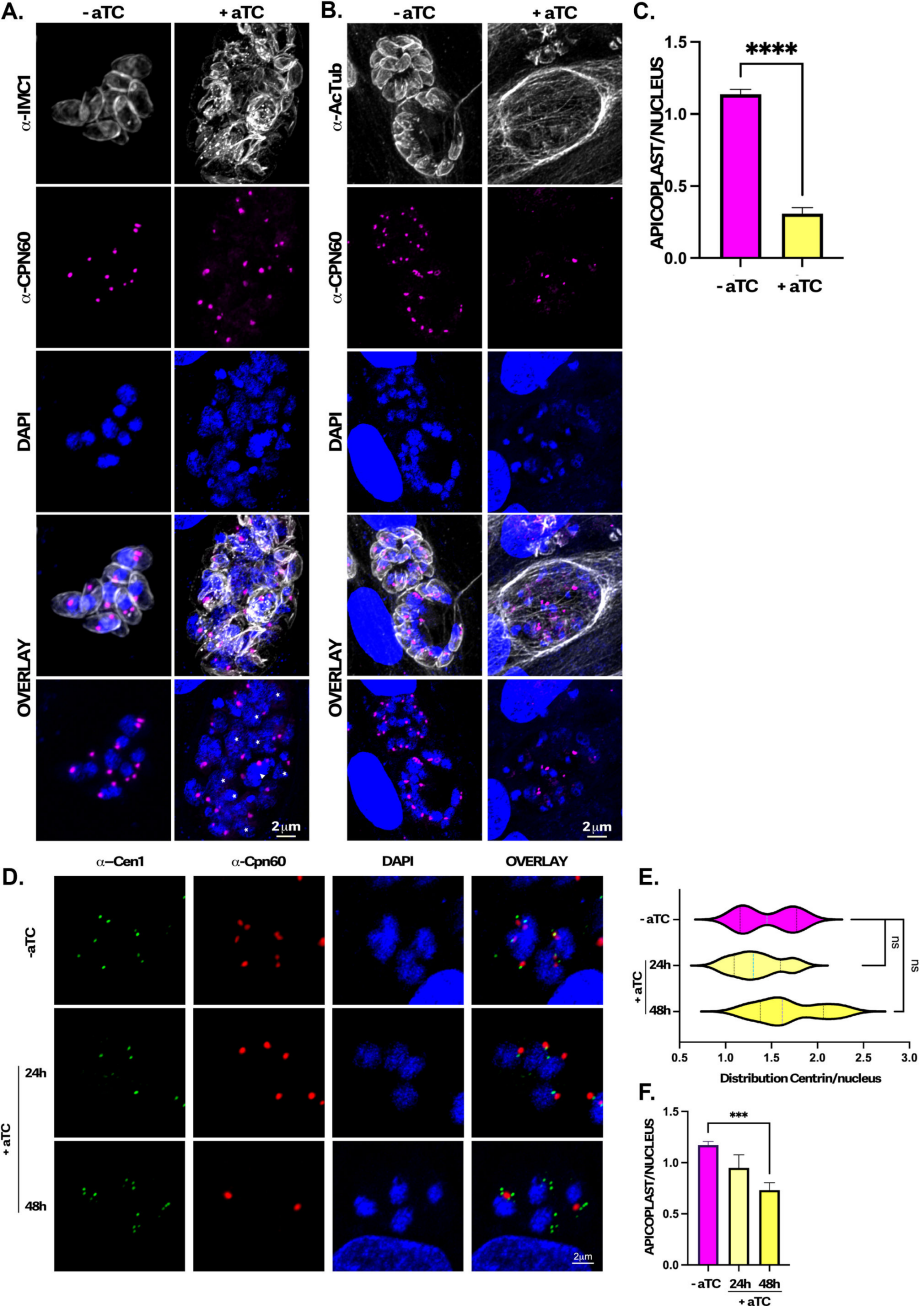
TgGSK knockdown parasites display a reduction in apicoplast material. (A) IFA of TATi-GSK parasites after 96 hours of aTC treatment stained for the apicoplast marker CPN60. IMC1 and DAPI staining are also used to visualize parasite structure and nuclear material. (B) IFA of TATi-GSK parasites after 96 hours of aTC treatment stained for the apicoplast marker CPN60. Acetylated tubulin and DAPI staining are also used to visualize parasite structure and nuclear material. (C) Quantification of the number of apicoplast foci per parasite nucleus in TgGSK knockdown versus control parasites. ****P < 0.0005, *n* = 3 experimental replicates, 100 total parasites quantified. (D) IFA of TATi-GSK parasites after 24 and 48 hours of aTC treatment stained for the centrosome marker centrin 1 and the apicoplast marker CPN60. DAPI staining was also used to visualize nuclear material. (E) Quantification of the distribution of centrin per nucleus in TgGSK knockdown at 24 and 48 hours vs control parasites. ns: no significance, *n* = 3 experimental replicates, 100 total parasites quantified. (F) Quantification of the number of apicoplast foci per parasite nucleus in TgGSK knockdown at 24 and 48 hours vs control parasites. ***P < 0.005, *n* = 3 experimental replicates, 100 total parasites quanitified.

The centrosome also coordinates the segregation of organelles other than the nucleus, including the apicoplast (18). Accordingly, we monitored apicoplast division and segregation (Fig. 6A and B). While parasites grown in the absence of aTC averaged around one apicoplast per parasite nucleus, aTC-treated parasites averaged one apicoplast for every four parasite nuclei (Fig. 6C). Therefore, it appears that, in addition to abnormal nuclear division and segregation, apicoplast division and segregation are also disrupted in TgGSK knockdown parasites.

To identify whether the apicoplast segregation defect is directly linked to the centrosome abnormalities, we analyzed earlier time points upon TgGSK depletion. We observed that upon acute depletion of TgGSK (24 hours), neither the number of centrosomes nor apicoplasts exhibit statistically significant differences compared to an untreated control (Fig. 6D through F). However, apicoplast segregation defects are noticeable at 24 hours, and more so at 48 hours (Fig. 6D). At this later point, treated parasites display 0.7 apicoplast/parasite, while the control remains at 1.2 apicoplast/parasite (Fig. 6F). By contrast, though a trend appears toward the phenotype observed at 96 hours post aTC addition, centrosome distribution is not statistically significantly different from the control at earlier time points (Fig. 6E). However, even in those parasites where apicoplast number is reduced, apicoplasts are consistently linked to the centrosome (Fig. 6D). These results suggest that the apicoplast distribution defects either precede, are more penetrant, or are mechanistically independent from those observed for the centrosome.

### Centrosomal, basal end, and splicing proteins show TgGSK-dependent phosphorylation

**Fig 7:**
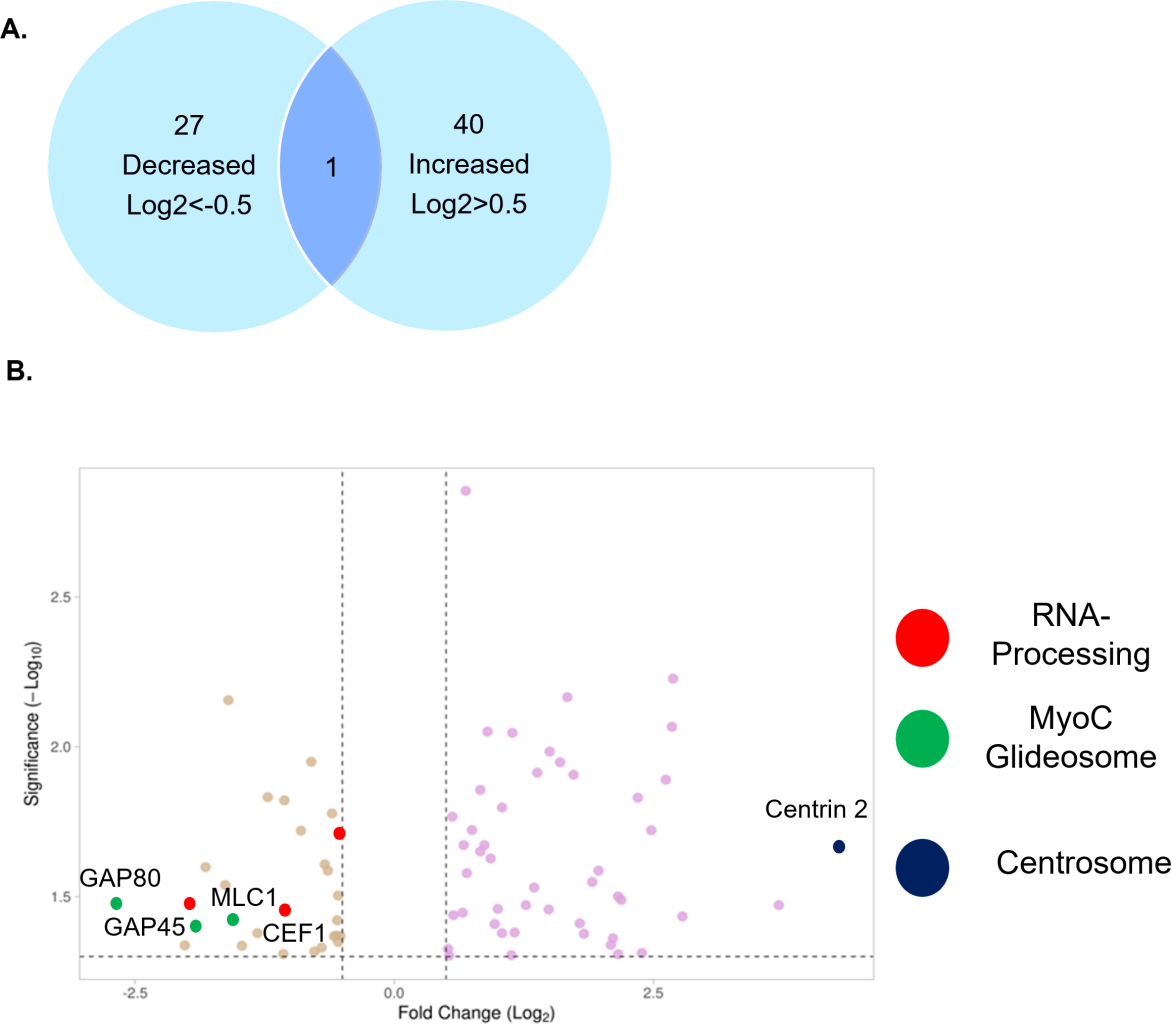
Global phosphoproteome analysis reveals TgGSK-dependent phosphorylation events. (A) Number of proteins that had increased or decreased phosphorylation after 24 hours of TgGSK knockdown. One protein had peptides with both increased and decreased phosphorylation. (B) Volcano plot of all differentially phosphorylated proteins with a log2 fold change >0.5 and *P* < 0.05. Splicing, myoC glideosome, and centrosome proteins are highlighted.

We next conducted global phosphoproteome analysis to identify TgGSK-dependent phosphorylation, revealing 27 proteins with decreased phosphorylation and 40 with increased phosphorylation in TgGSK knockdown parasites (log2FC > 0.5, *P* < 0.05) (Fig. 7A; Data set S1). Pathway analysis using ToxoDB and StringDB highlighted several enriched pathways. Notably, three of the proteins with decreased phosphorylation were RNA-binding or splicing proteins, and three were part of the MyoC glideosome complex (Fig. 7B). Interestingly, five of these six proteins exhibited differential phosphorylation at an S/TXXXS/T motif typical of direct GSK substrates (19). In addition, the protein that had the highest increase in phosphorylation in the knockdown was centrin 2 (Fig. 7B). It was also found that 18% of hypothetical proteins with TgGSK-dependent phosphorylation were linked to RNA metabolism (Fig. S1). Overall, phosphoproteome analysis suggests that TgGSK might influence the regulation of proteins related to the centrosomes, basal complex, and splicing.

### TgGSK interacts with and is regulated by the GCN5b complex

**Fig 8:**
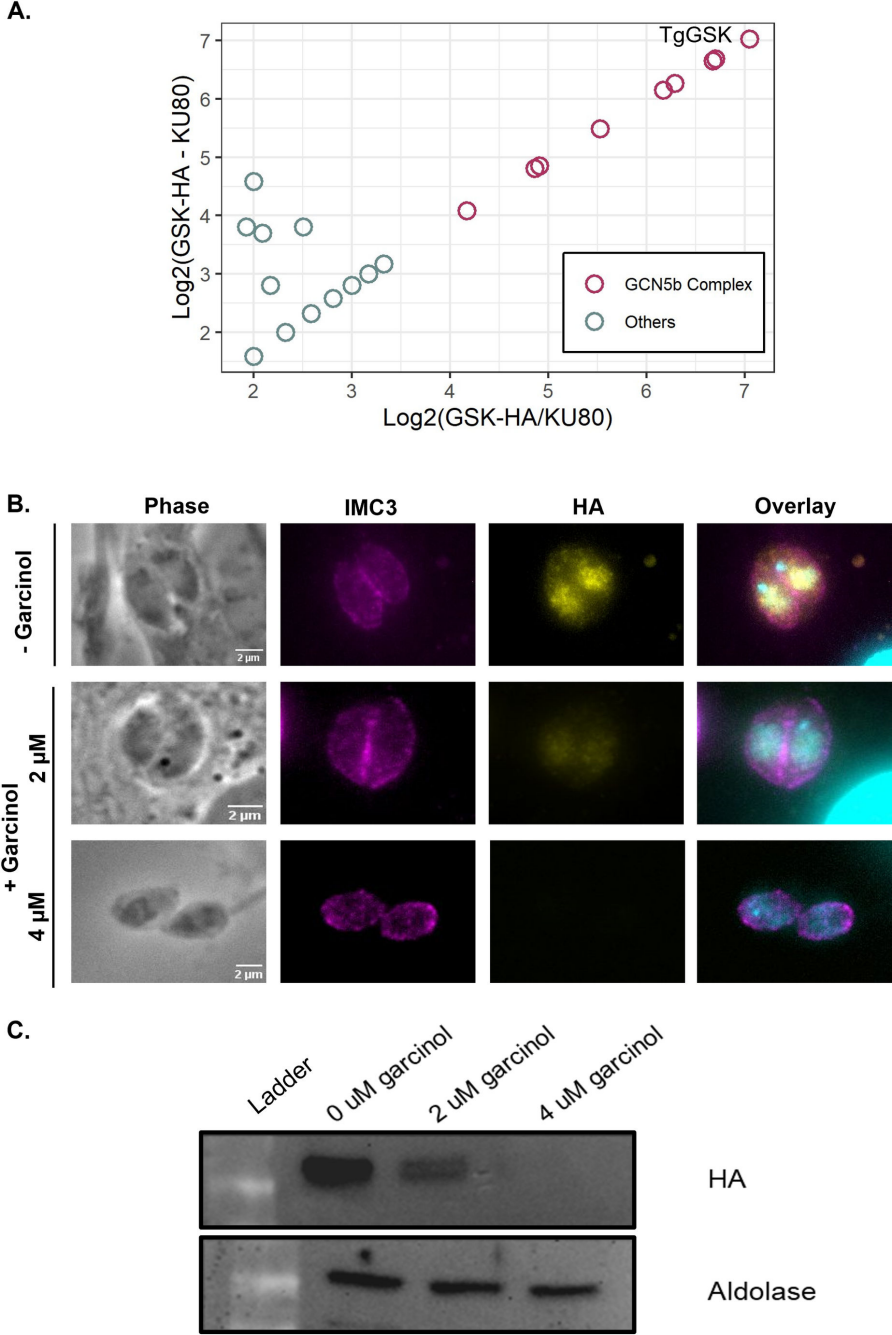
Acetylation by GCN5b stabilizes TgGSK. (A) Plot of TgGSK immunoprecipitation data. More significant interactors are toward the upper right of the plot. Points in red are members of the GCN5b complex. (B) IFA of non-dividing GSK.3xHA parasites treated with 0, 2, or 4 µM garcinol for 18 hours. Staining was done for IMC3, HA, and DAPI to visualize IMC, TgGSK, and nuclear material, respectively. (C) Western blot analysis of TgGSK protein levels after 18 hours of garcinol treatment. The cytosolic protein aldolase was used as a control.

To further dissect the multiple putative roles of TgGSK, we performed immunoprecipitation followed by mass spectrometry to identify interacting partners. Interestingly, eight of the top nine significant TgGSK interacting proteins were nuclear, with all eight of them being members of the previously characterized GCN5b complex (Fig. 8A; Table 1; Data set S2). Key components of this complex include the lysine acetyltransferase GCN5b, the transcription factors AP2IX-7 and AP2X-8, and ADA2a (20).

**TABLE 1:** TgGSK interactors. ID number and annotation of putative interactors of TgGSK identified in both IPs performed with a ratio of experimental over control higher than 5. Members of the GCN5b complex are shown in gray

ID number	Protein annotation
TgGT1_214960	AP2X-8
TgGT1_217050	ADA2a
TgGT1_226620	Hypothetical protein
TgGT1_229640	Apical cap protein AC8
TgGT1_241850	Hypothetical protein
TgGT1_243440	GCN5b
TgGT1_274180	Hypothetical protein
TgGT1_280590	Hypothetical protein
TgGT1_290630	AP2IX-7

While TgGSK may regulate members of the GCN5b complex via phosphorylation, we did not identify any of these proteins in the TgGSK-dependent phosphoproteome. Instead, we investigated whether the GCN5b complex may regulate TgGSK. We treated parasites with garcinol, an acetyltransferase inhibitor shown to inhibit the histone acetylation activity of GCN5b in *Toxoplasma* (21). We treated GSK.3xHA parasites with 0, 2, or 4 µM of garcinol overnight and monitored TgGSK localization by IFA. While the localization pattern remained unchanged, the overall TgGSK signal decreased in a dose-dependent manner, disappearing at 4 µM (Fig. 8B). Western blot analysis confirmed these findings, showing reduced TgGSK levels at 2 µM and complete absence at 4 µM relative to aldolase, which was unaffected at all concentrations (Fig. 8C). Previous data indicated that TgGSK transcript levels were unchanged by garcinol, suggesting that GCN5b regulates TgGSK at the protein level (21). Therefore, it appears that TgGSK’s protein expression or stability might be regulated by GCN5b.

### TgGSK knockdown causes differential transcription and splicing

**Fig 9:**
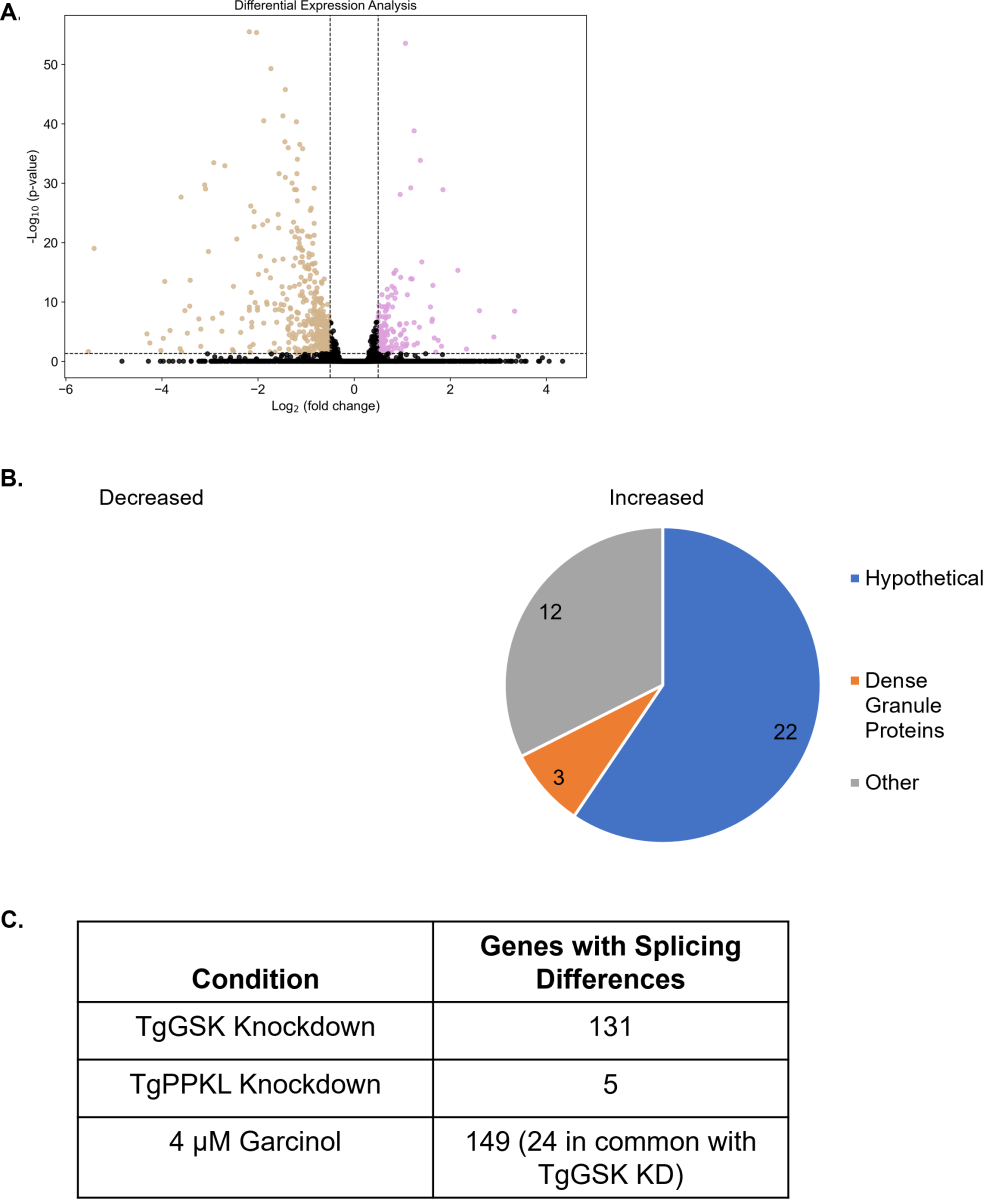
Global transcriptome analysis of TgGSK knockdown parasites. (A) Volcano plot showing all differentially transcribed genes with a log2 fold change >0.5 and *P* < 0.05 after 18 hours of TgGSK knockdown. (B) Biological processes with enriched transcriptome changes as identified by ToxoDB and StringDB. (C) Genes that were differentially spliced after TgGSK knockdown, TgPPKL knockdown, and treatment with 4 µM garcinol.

As TgGSK is in a complex with the transcription factors AP2IX-7 and AP2X-8, we investigated the effect of TgGSK knockdown on global transcription. For this purpose, we performed RNAseq of the TATi-GSK.3xHA strain grown for 18 hours with or without aTC. This time point was chosen to capture transcriptional changes before parasite death, as TgGSK protein was still present, albeit at reduced levels. We identified 405 downregulated and 157 upregulated genes upon TgGSK knockdown (log2FC > 0.5, *P* < 0.05) (Fig. 9A; Data set S3). Most of the differentially regulated genes were hypothetical or did not fall into any specific enriched pathway (Fig. 9B). Notably, comparing these genes to those known to be bound by the GCN5b complex showed no enrichment for GCN5b-regulated genes (20).

As we identified several RNA processing proteins in the TgGSK-dependent phosphoproteome, we investigated the RNAseq data for splicing variants. Interestingly, we found that 131 genes had exon differences in TgGSK knockdown parasites compared to parental (Fig. 9C). As a control, we analyzed exon differences in TgPPKL knockdown parasites, which is also essential and causes division-related defects, and found that only five genes were differentially spliced (Fig. 9C). In addition, since garcinol treatment resulted in loss of TgGSK, we mined previously published transcriptomic data from garcinol-treated parasites (21) to assess the effects on splicing. This analysis revealed 149 differentially spliced transcripts in parasites treated with 4 µM garcinol (Fig. 9C). Interestingly, 24 of these overlapped with transcripts that were differentially spliced in the TgGSK knockdown (Fig. 9C). Overall, these data suggest that TgGSK plays a role in proper splicing.

## DISCUSSION

Glycogen synthase kinases (GSKs) are serine/threonine kinases found in numerous organisms (22). Mammals typically have two GSKs, GSK3α and GSK3β, which regulate cellular processes like proliferation, migration, glucose metabolism, and apoptosis (23). In contrast, plants have 10 GSKs divided into 4 major groups, involved in growth, development, and stress responses (24). *Toxoplasma gondii*, like other Apicomplexa, has two putative GSKs (TGGT1_265330 and TGGT1_266910), which are largely uncharacterized. This study focused on TGGT1_265330, referred to as TgGSK, revealing that its localization depends on the cell cycle and that it affects daughter cell formation, nuclear segregation, centrosome dynamics, apicoplast dynamics, and splicing. The broad range of functions affected by TgGSK is consistent with the often-pleiotropic characteristics of GSKs.

The centrosome is an essential component of endodyogeny, with each of the two daughter parasites forming around a centrosome (25). It nucleates spindle microtubules during mitosis and organizes components for nuclear fission and organelle segregation during cytokinesis. The apicoplast, a non-photosynthetic plastid, elongates and interacts with centrosomes before fission, which ensures its proper orientation (25). Using UExM, we visualized TgGSK at the centrosomes. Knockdown of TgGSK caused defects in centrosome number and splitting, which could be leading to the defects we see in nuclear and apicoplast segregation. We also observed TgGSK-dependent phosphorylation of centrin 2, a protein that is localized to the centrosomes and has been shown to be essential for parasite division and centrosome splitting (26). In our study, centrin 2 phosphorylation was increased during TgGSK knockdown, suggesting that it is not a TgGSK substrate and that its regulation of centrin 2 is indirect. Interestingly, centrin 2 has also been shown to be localized to the basal body (26). As we also detect TgGSK within the basal end of the parasite, it is unclear where the functional relationship between these two proteins occurs.

A role for GSKs in centrosome regulation is also observed in other organisms. Inhibition of GSK3β in human cancer cells results in centrosome dysregulation and abnormal mitosis (27). Similarly, in HeLa cells, GSK3β plays a role in organizing microtubule arrays derived from the centrosomes (28). Though recruitment of the mitotic spindle binding protein EB1 is not altered in the absence of TgGSK, further studies are needed to determine if centrosomal proteins or mitotic factors are negatively affected.

Intriguingly, we found that the phosphorylation state of three RNA-binding proteins (TGGT1_264610, TGGT1_275480, and TGGT1_304630) changes in the absence of TgGSK. TGGT1_264610 and TGGT1_304630 are putative RNA-binding proteins related to splicing, while TGGT1_275480 is homologous to the pre-splicing factor CEF1. TGGT1_264610 and TGGT1_275480 are fitness conferring with CRISPR scores of −3.94 and −4.24, respectively. They also each have nine phosphorylation sites. TGGT1_304630 is dispensable, with a CRISPR score of −0.44, and has dozens of phosphorylation sites. All three contain the S/TXXXS/T motif, suggesting they may be direct TgGSK substrates (20). In mouse embryonic stem cells inhibition of GSK3 altered the splicing of 188 mRNAs (29). In this same study, GSK was also shown to interact with multiple SR family splicing proteins (29). In human T-cells, GSK3 phosphorylation of PSF regulates CD45 alternative splicing (30). Although the plant GSK homolog BIN2 lacks direct evidence for splicing involvement, its signaling network includes 13 RNA processing proteins (10).

A key finding of our study is the potential regulation of TgGSK by the lysine acetyltransferase GCN5b. We observed that TgGSK interacts with a well-characterized GCN5b-containing complex. The essential GCN5b complex is in the nucleus, where it performs its primary function of acetylating histones (20). GCN5b exists in two distinct complexes with differing AP2 putative transcription factors (31). Our results indicate that TgGSK interacts with the complex that includes AP2X-8 and AP2IX-7 (Table 1). Interestingly, previous characterization of the *Toxoplasma* GCN5b complexes identified TgGSK as an interactor, further validating this interaction (31).

**Fig 10:**
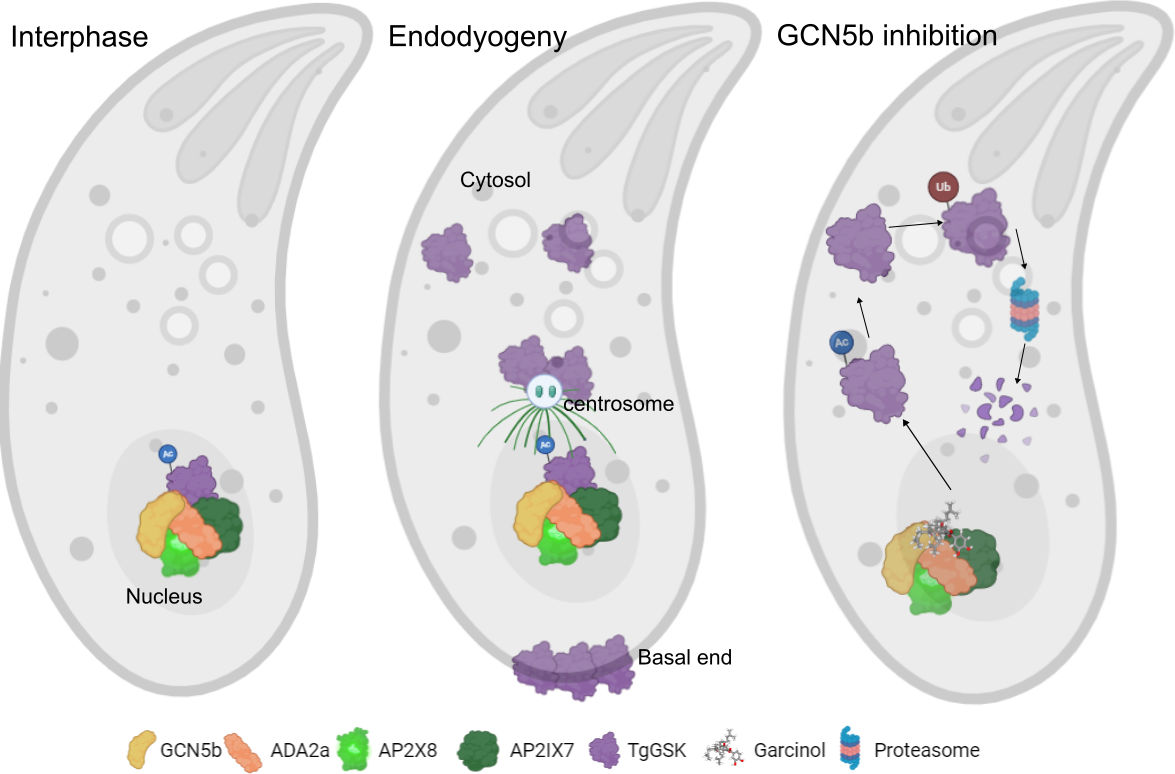
Model of the regulation and function of TgGSK. A preliminary model of TgGSK in non-dividing and dividing parasites. Image created using Biorender.

Interestingly, we also observed a specific loss of TgGSK upon GCN5b inhibition by garcinol. This result brings up the possibility that GCN5b regulates TgGSK via acetylation. While acetylation of histones is canonical, many studies have shown acetylation of non-histone proteins, which can impact their folding and stability (32, 33). Interestingly, a global acetylome study in *Toxoplasma* identified acetylation on TgGSK at residue K13 in extracellular parasites, a residue that has also been shown to be ubiquitinated (34). Cross-talk between acetylation and ubiquitination of the same lysine is well-characterized and is particularly important in protein stability, where lysine acetylation can block proteasome-mediated degradation (35). We propose that TgGSK regulation could involve its acetylation within the nucleus by GCN5b at residue K13 before being trafficked to the cytosol and centrosomes in preparation for cell division. Once division has finished, TgGSK could be deacetylated and ubiquitinated at K13, causing its degradation (Fig. 10). Interestingly, in Alzheimer’s disease, acetylation of GSK3β at residue K15 (the equivalent of TgGSK K13) leads to the over-activation of the kinase, leading to the hyperphosphorylation of tau (36). Attempts to mutate K13 to either arginine (acetylation null) or glutamine (acetylation mimic) led to parasite death suggesting that this amino acid is important for function (data not shown).

Overall, the findings in this study highlight the various roles of TgGSK in *Toxoplasma*. As BIN2 and mammalian GSKs have been shown to have hundreds of substrates with many different biological functions, the broad range of possible TgGSK functions uncovered here is not surprising. While likely roles for TgGSK in the centrosome and splicing have been identified through this work, further studies are warranted to understand the mechanistic underpinning of these functions. The critical importance of this essential kinase underscores its strong potential as a target for antiparasitic intervention.

## MATERIALS AND METHODS

### Parasite strains and reagents

All parasite strains used in this study derived from the strain RH lacking HXGPRT and Ku80 (RH∆*ku80*∆*hxgprt*) (37). Parasites were maintained in human foreskin fibroblasts (HFF) with standard growth medium as previously described (38).

### Phylogenetic analysis

GSKs from 21 different organisms from all eukarya kingdoms were selected. Accession numbers are *Arabidopsis thaliana* (AtBIN2_Q39011, AtGSK3_NP_973771), *Oryza sativ japonica* (GSK1_ORYSJ_Q9LWN0, GSK2_ORYSJ_Q60EZ2), *Symbiodinium* sp. (GSK3B_Symbiodinium_CAE7846410), *Schizosaccharomyces pombe* (SchpGSK3_CAA22609), *Aspergillus melleus* (AmGSK3_XP_045946591), *Drosophila melanogaster* (Dm_shaggyGSKisoforml_NP_996337), *Homo sapiens* (GSK3b_P49841; GSK3a_KAI4042991), *Danio rerio* (DrGSK3B_XP_009302769), *Leishmania major* (LmjGSK3B_LmjF.18.0270, LmjGSK3A_LmjF.22.0490), *Giardia muris* (GmGSK_GMRT_14462, GmGSK_GMRT_15846), *Neospora caninum* (NCLIV_039150; Ncaninum_LIV_000226500), *Plasmodium falciparum* (PF3D7_1316000; PF3D7_0312400), and *Toxoplasma gondii* (GSKβ _TGME49_265330 aka TgGSK; GSK3⍺_TGME49_266910). Sequences were aligned using Clustal W, and the bootstrap neighbor joining tree was constructed using the Blosum62 algorim. The phylogenetic tree was visualized with Evolveview (39).

### Generation of parasite lines

All primers used are listed in Table S1. To add a hemagglutinin (HA) epitope tag to the endogenous TgGSK gene, we amplified the 3×HA-DHFR (40) amplicon from LIC-3xHA-DHFR using primers that allowed for recombination at the 5′ end with sequences immediately upstream of the stop codon and at the 3′ end with sequences after the Cas9 cutting site. We modified pSag1-Cas9-U6-sgUPRT (41) using Q5 Site-Directed Mutagenesis Kit (NEB) to replace the UPRT guide RNA sequence with a TgGSK targeting guide. This plasmid and the amplicon were transfected into parasites using a Lonza Nucleofector. Transfected parasites were selected and cloned as previously described (42).

To generate the TgGSK conditional knockdown strain, we used a CRISPR-Cas9 mediated strategy to introduce a tet-OFF cassette upstream of the TgGSK start codon (43). Specifically, sequences for a gRNA targeting *TgGSK* downstream of the start codon were cloned into pSag1-Cas9-pU6-sgUPRT (41) using the Q5 mutagenesis kit. The tet-OFF cassette was amplified from pT8TATi-HXGPRT-tetO7S1 (43). Two micrograms of pSag1-Cas9-U6-sgGSK-KD and the PCR amplicon were transfected into the TgGSK-3xHA parasites using a Lonza nucleofector. After selection with 50 mg/mL mycophenolic acid and xanthine, transfected parasites were cloned. Integration of the tet-OFF cassette was validated by PCR, and the strain was named TATi-GSK.3xHA. To induce knockdown of GSK, this strain was grown in 1 µM of anhydrotetracycline (ATC) from Sigma Aldrich.

### Plaque assays

Standard plaque assays were performed as previously described (38). Briefly, 500 parasites of each strain were seeded into host cell monolayers grown in 12-well plates, and cultures were grown for 6 days. Cultures were fixed with methanol and stained with crystal violet, and plaques were quantified by averaging the percentage of cleared area in each of three wells of each condition over three experimental replicates (38).

### Immunofluorescence assays

Immunofluorescence assays (IFAs) were performed as previously described (38). The primary antibodies used include rabbit anti-HA (Cell Signaling Technologies), rat anti-IMC3 (provided by Dr. Marc-Jan Gubbels, Boston College), mouse anti-centrin 1 (Cell Signaling Technologies), and mouse anti-acetylated tubulin (Sigma Aldrich) at a concentration of 1:1,000; guinea pig anti-TgEB1 (provided by Dr. Marc-Jan Gubbels, Boston College) at a concentration of 1:3,000; rabbit anti-TgH2Bz (provided by Dr. Laura Vanagas and Dr. Sergio Angel, INTECH-Chascomus) at a concentration of 1:500; and rabbit anti-Cpn60 (provided by Dr. Erica Dos Santos Martins, UFMG) at a concentration of 1:300. Secondary antibodies used were conjugated to Alexa Fluor 405, 488, 594, and 647 (Invitrogen) at 1:2,000. For images in Fig. 2B and C, 3B, 4A, and 9A, a Nikon Eclipse E100080i microscope with NIS Elements AR 3.0 software was used. For images in Fig. 4D, 5A, and 6A, a Zeiss LSM800 confocal microscope with Zeiss ZEN blue v2.0 and Huygens Professional v19.10.0p2 software was used for deconvolution. ImageJ was used for all analysis.

Quantification of the GSK-HA signal in the nucleus and cytoplasm was performed by imaging 20 non-dividing parasites and 20 in late division, all in different vacuoles, using a Nikon Eclipse E100080i microscope with NIS Elements AR 3.0 software. Using ImageJ, fluorescent intensity was measured along lines drawn in the cytosol and the nucleus of each parasite, and the ratio of nuclear to cytosolic intensity was calculated.

### Ultrastructure expansion microscopy

UExM was performed as described previously with the modification of parasites being grown on HFF monolayers (44). Primary antibodies used include rabbit anti-*Toxoplasma* tubulin (provided by Dr. Michael Reese, UT Southwestern), rabbit anti-centrin 1, and mouse anti-HA at a concentration of 1:500. DRAQ5 (1:500) and NHS ester (1:250) were also used. Secondary antibodies were as for IFA. Imaging was performed using a Zeiss LSM900 microscope with Zeiss ZEN Blue software before image analysis using ImageJ.

### Western blots

Western blots were performed as described previously (38). The primary antibodies used include rabbit anti-HA (Cell Signaling), mouse anti-Sag1 (Invitrogen), and mouse anti-aldolase, all at 1:5,000. The secondary antibodies were HRP-labeled anti-mouse and anti-rabbit IgG, at 1:10,000. Blots were imaged with a ProteinSimple system.

### Immunoprecipitation

Immunoprecipitation was performed as previously described with some modifications (42). For immunoprecipitation from whole-parasite lysate, intracellular parasites were harvested after 18 hours of growth. Cells were lysed at 4°C for 1 hour in 500 µL RIPA lysis buffer supplemented with 5 µL protease and phosphatase inhibitor cocktail (Thermo Scientific). Samples were sonicated three times and centrifuged at maximum speed for 10 minutes at 4°C. Supernatants were incubated with mouse IgG magnetic beads for 1 hour at 4°C for pre-cleaning and then with rabbit HA magnetic beads (Thermo Scientific) overnight at 4°C. After washing with RIPA lysis buffer and PBS, the beads were submitted to the IUSM Proteomics Core facility for liquid chromatography coupled to tandem mass spectrometry (LC/MS-MS) analysis.

### Global transcriptomic analysis

TATi-GSK.3xHA parasites were grown for 18 hours with or without ATC and harvested with host cells by scraping in cold PBS, followed by centrifugation at 2,000 rcf for 5 minutes at 4°C. The pellet was passed through a 27-gauge needle in 10 mL PBS to release parasites. After centrifugation, the pellet was treated with 1 mL TRIZOL for 5 minutes at room temperature before extracting RNA with 200 µL of chloroform and centrifuging at 12,000 rcf for 15 minutes at 4°C. The aqueous phase was again treated with 500 µL of chloroform and centrifuged to extract RNA. The aqueous phase was mixed with 500 µL of isopropanol and incubated at room temperature for 10 minutes before centrifuging at 12,000 rcf for 10 minutes. RNA was washed with 1 mL 75% ethanol and centrifuged at 7,500 rcf for 5 minutes. The pellet was air dried and resuspended in 50 µL of nuclease-free water. Samples were sent to AZENTA for library construction and sequencing utilizing Illumina Next Generation Sequencing technology. For each sample, ~30 M 2 × 150 bp pair-end reads were obtained. The GALAXY online platform was used for data analysis. The quality of the sequencing data was checked using FastQC, and adapter sequences were trimmed using Trim Galore. Hisat2 and htseq-count were separately employed to map reads to the genome and transcript reads. DEseq2 was used to analyze differential gene expression and DEXseq to analyze splicing. Pathway analysis was done through ToxoDB and StringDB.

### Global phosphoproteomics

TATi-GSK.3xHA parasites were grown for 24 hours with or without ATC. and harvested as for RNAseq. The parasite-containing pellets were flash-frozen in liquid nitrogen and stored at −80°C before being sent to the IUSM Proteomics Core for global phosphoproteome analysis as described (7). Protein function and pathway information were determined using ToxoDB and StringDB.

### Garcinol assays

GSK.3xHA parasites were seeded simultaneously with 2 or 4 µM Garcinol (BOC Sciences) in 1% serum media for 18 hours. Immunofluorescence analysis and western blots were done as described above.

## SUPPLEMENTAL MATERIAL

Data set S1 msphere.00111-25-s0001.xlsx Phosphoproteome raw data.

Data set S2 msphere.00111-25-s0002.xlsx Immunoprecipitation raw data.

Data set S3 msphere.00111-25-s0003.xlsx RNAseq raw data.

Supplemental material msphere.00111-25-s0004.pdf Table S1, Figure S1, and Figure S2.

Supplemental legends msphere.00111-25-s0005.docx Legends for supplemental figures, table, and data sets.

ASM does not own the copyrights to Supplemental Material that may be linked to, or accessed through, an article. The authors have granted ASM a non-exclusive, world-wide license to publish the Supplemental Material files. Please contact the corresponding author directly for reuse.
