# Supplementary material for: The essential kinase TgGSK regulates centrosome segregation and endodyogeny in Toxoplasma gondii
Source: mSphere. 2025 Mar 28;10(4):e00111-25. doi: 10.1128/msphere.00111-25 (PMC12039231; doi:10.1128/msphere.00111-25)
Supplement: Supplemental material — Table S1, Figure S1, and Figure S2. [file msphere.00111-25-s0004.pdf]

Purpose	Name	Sequence
Generate pSag1-Cas9-U6-sgGSK-TG-HXG CRSPR/Cas9 plasmid for endogeneous tagging	GSK-tg-sgRNA.For	GTCTTTTTTTGTTTTAGAGCTAGAAATAGC
	GSK-tg-sgRNA.Rev	CGACAGCTGCAACTTGACATCCCCATTTAC
Amplify 3xHA-DHFR cassette from the plasmid pLIC-3xHA-DHFR	GSK-TG-insert.For	ATGTATTCCGAAGCATATCGCCAGTGCAAACAACC GTGGCTTAATTAAAATTGGAAGTGGAGG
	GSK-TG-insert.Rev	AGCATAAGAGAAGCTCCCCATCCCTAGTAGGTGTA GGGAGGTTTTCCCAGTCACGACG
Generate pSag1-Cas9-U6-sgGSK-TATI-HXG CRSPR/Cas9 plasmid for endogeneous tagging	GSK-TATI-sgRNA.For	AAGAAGGGGTGTTTTAGAGCTAGAAATAGC
	GSK-TATI-sgRNA.Rev	TCCTTCGTCCAACTTGACATCCCCATTTAC
Amplify TATi cassette from the plasmd 5'COR-pT8TATi1-HX-tetO7S1mycNtCOR.dna	GSK-TATI-insert.For	CACTCATCTTTTCCTGGCCTTTGTCGAGAAGGCAG AAGTCTCTTCTCATGTTTGCGGATCCG
	GSK-TATI-insert.Rev	CTACTCTTCTGAGCAGCTGCGGGATCGTACTGCG GGTCCGGCATTTTGATATCCCTAGGAATTCACTC

Supplemental table 1. Primers used in this work. Sequences are 5’ to 3’.

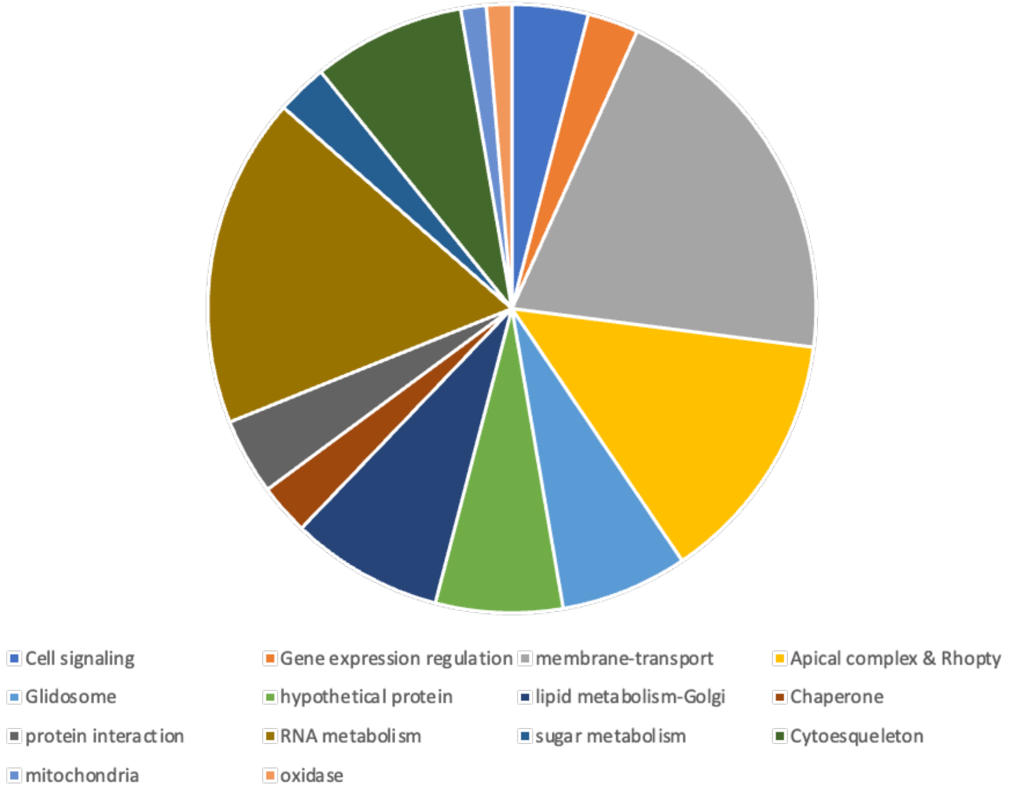

Supplemental figure S1. Classification of hypothetical proteins with TgGSK-dependent phosphopeptides. Hypothetical proteins with peptides differentially phosphorylated in the knockdown vs the parental were analyzed based on annotations in the *Toxoplasma* genome database or homology to proteins in other Apicomplexan species. Additionally, the protein domains were analyzed based on known conserved functions. Some of these proteins remained classified as hypothetical.

Blot for figure 2A:

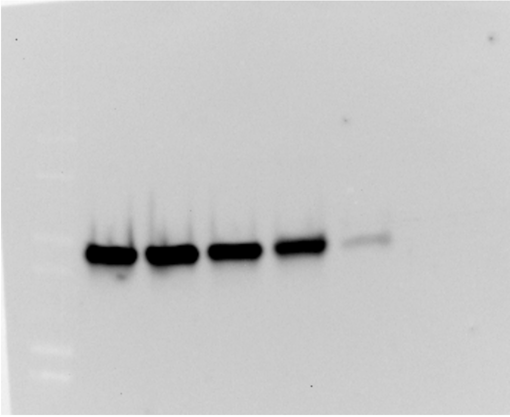

Lane 1: Ladder
Lanes 2-4: Intracellular TgGSK
Lanes 4-5: Extracellular TgGSK

Blot for figure 3B:

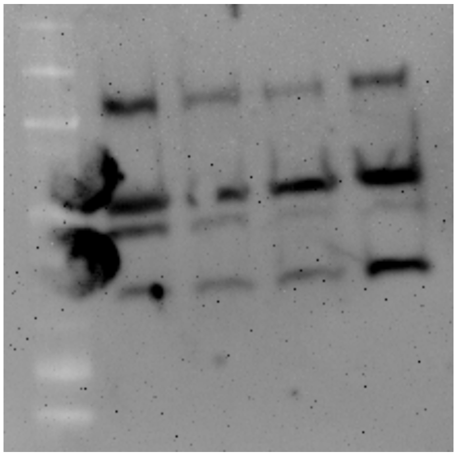

Lane 1: Ladder
Lane 2: No aTC
Lane 3: 24 hours aTC
Lane 4: 42 hours aTC
Lane 5: 72 hours aTC

Figure 8C:

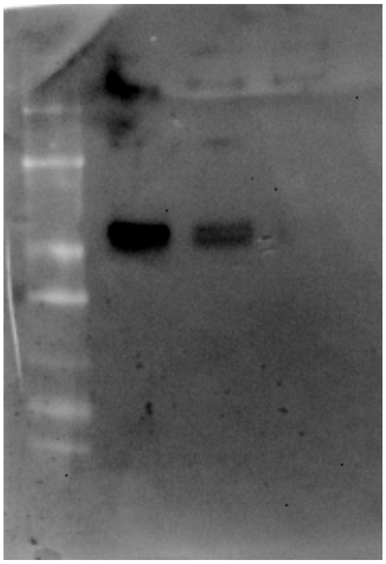

Lane 1: Ladder
Lane 2: No Garcinol
Lane 3: 2 µM Garcinol
Lane 4: 4 µM Garcinol

Supplemental figure S2. Full Western blots
