# Supplementary material for: The essential kinase TgGSK regulates centrosome segregation and endodyogeny in Toxoplasma gondii
Source: mSphere. 2025 Mar 28;10(4):e00111-25. doi: 10.1128/msphere.00111-25 (PMC12039231; doi:10.1128/msphere.00111-25)
Supplement: Supplemental legends — Legends for supplemental figures, table, and data sets. [file msphere.00111-25-s0005.docx]

**Legends for supplemental materials**

**Supplemental table 1.** Primers used in this work. Sequences are 5’ to 3’.

**Supplemental figure S1. Classification of hypothetical proteins with TgGSK-dependent phosphopeptides.** Hypothetical proteins with peptides differentially phosphorylated in the knockdown vs the parental were analyzed based on annotations in the *Toxoplasma* genome database or homology to proteins in other Apicomplexan species. Additionally, the protein domains were analyzed based on known conserved functions. Some of these proteins remained classified as hypothetical.

**Supplemental figure S2.** Full Western blots

**Supplemental dataset 1.** Raw data from phosphoproteome of conditional knockdown strain with and without ATc.

**Supplemental dataset 2.** Raw data from immunoprecipitation experiments.

**Supplemental dataset 1.** Raw data from RNAseq of conditional knockdown strain with and without ATc.
